# Ferroelectric Oxide Nanocomposites with Trimodal Pore Structure for High Photocatalytic Performance

**DOI:** 10.1007/s40820-019-0268-y

**Published:** 2019-05-04

**Authors:** Tingting Xu, Xuan Liu, Shulan Wang, Li Li

**Affiliations:** 1 Department of Chemistry, School of Science, Northeastern University, Shenyang, 110819 People’s Republic of China; 2 School of Metallurgy, Northeastern University, Shenyang, 110819 People’s Republic of China

**Keywords:** Ferroelectric photocatalysis, Piezoelectric, Hierarchical pores, Photocatalytic hydrogen production, Triple-junction

## Abstract

An effective method to improve the photocatalytic performances of powder catalysts is to use the internal electric field from ferroelectrics to separate photogenerated charge carriers. The design and engineering of a complex hetero-junction with a hierarchical pore structure is highly desirable for the efficient application of ferroelectric materials in photocatalysis. Here, we present a novel strategy using two templates to fabricate PbTiO_3_/TiO_2_/carbon (PTC) nanocomposites with a tunable microstructure. A hard SiO_2_ template combined with an ice template followed by an appropriate pyrolysis procedure introduced trimodal (micro-, meso-, macro-) porosity. The as-prepared PTC nanocomposites with optimal mass ratio exhibited excellent photocatalytic and photoelectrochemical performances. PbTiO_3_/TiO_2_ annealed at 900 °C (PTC-900) showed a MB degradation rate of 0.21 and 0.021 min^−1^ under UV and visible light irradiation, which are, respectively, 7.2 and 3 times those of pure PbTiO_3_. The photocurrent density of the composite catalyst is 1.48 mA cm^−2^ at the potential of 1.0 V versus saturated calomel electrode, and the rates of hydrogen generation of PTC-900 are as high as 2360 and 9.6 μmol h^−1^ g^−1^ under UV and visible light irradiation, respectively. More importantly, the simultaneous application of ultrasound-induced mechanical waves further improved the photocatalytic reactivity. This work serves to improve understanding on the design of ferroelectric/piezoelectric photocatalysts with a hierarchical pore structure and also proposes a widely applicable strategy for the fabrication of high-performance micro–nano/nano–nano structures.

**Electronic supplementary material:**

The online version of this article (10.1007/s40820-019-0268-y) contains supplementary material, which is available to authorized users.

## Highlights

- PbTiO_3_/TiO_2_@carbon (PTC) composites with trimodal pore distribution were synthesized by a dual-template method. Localized quasi-1D nanoneedle morphology was formed that can promote charge separation.
- The as-prepared PTC nanocomposites with optimal mass ratio exhibited excellent photocatalytic and photoelectrochemical performances.

**Electronic supplementary material:**

The online version of this article (10.1007/s40820-019-0268-y) contains supplementary material, which is available to authorized users.

## Graphical abstract

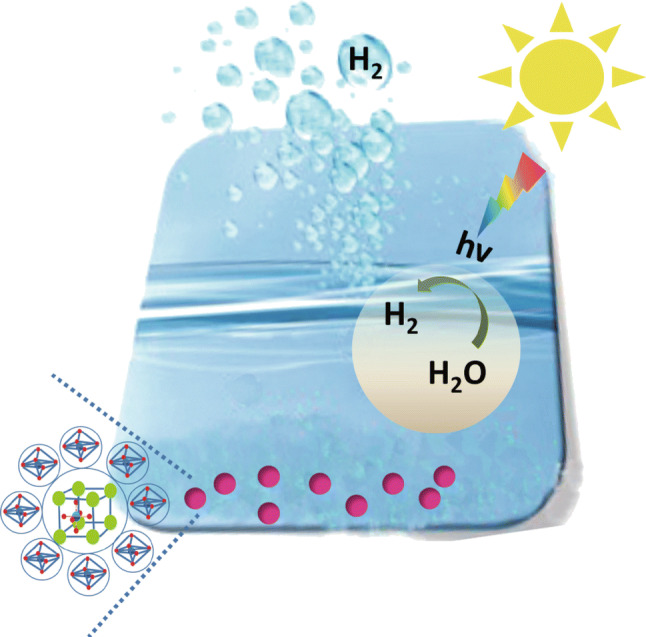

## Introduction

Semiconductor photocatalysis is regarded as one of the most attractive sustainable technologies to address global energy and environmental challenges [1]. Developing efficient strategies to separate photogenerated charge carriers is highly desirable to improve solar energy conversion efficiency in powder photocatalysts [2]. The built-in electric field in different interfaces as *p*-*n* junctions [3], polymorph junctions [4], or polar interfaces [5] guides electrons and holes to move in different directions and prevents their recombination. Ferroelectric perovskite materials with ABO_3_ structure, such as BaTiO_3_ and PbTiO_3_, have self-polarization in different domains induced by the migration of cations at B sites leading to non-centrosymmetric positive and negative charges [6, 7]. The dipolar field induced by ferroelectricity has been shown to enable the directional separation of charge carriers [3, 4, 8–12]. Consequently, the development of ferroelectrics-based semiconductor photocatalysts is viewed as a promising strategy to overcome the shortage of low separation efficiency of charge carriers [13].

High stability in diverse environmental conditions is important for the practical application of powder photocatalysts. However, most ternary oxides are easily corroded by acid or alkali [14]. In view of its remarkable chemical stability, TiO_2_ has been coupled with ferroelectric materials [3, 15, 16]. In addition, it has been reported that the presence of TiO_2_ helps to lower the Schottky barrier between the co-catalyst used in hydrogen production and the photocatalytic light absorber [11]. However, ferroelectrics–semiconductor composites have the drawback of having low electrical conductivity [17]. Loading carbon can improve the conductivity of composite materials, and carbon can also serve as an electron acceptor to promote charge separation. Moreover, carbon acts as a photosensitizer to extend the absorption range of the composite photocatalyst and its high surface area allows carbon to full loading of photocatalysts [18]. In other words, the photocatalytic reaction is primarily limited by three steps, namely light absorption, photogenerated charge carrier separation, and surface reaction. From the microstructure point of view, an ideal photocatalyst should have a large surface area with an appropriate pore size distribution at different length scales. Some previous studies have demonstrated the advantages of synergistic effects in samples with multimodal pore size distribution over those with uniformly sized pores [19]. Macropores lead to increased light scattering and enhance photon absorption at the inner surface of oxides [20–22]. Mesopores provide abundant active sites for surface redox reactions between the solution and the photocatalyst and decrease the distance travelled by charge carriers [23], and micropores trap ions present in solution to promote surface reactions [21, 22]. However, building a hierarchical porous structure with pore sizes extending over the three length scales in triple semiconductor junctions still remains a challenge. Furthermore, there are very few reports on carbon containing ferroelectric photocatalysts.

Herein, we report for the first time, the design and synthesis of a hierarchical ferroelectrics-based porous composite PbTiO_3_/TiO_2_@C (PTC) with micro-, meso-, and macropores via a simple and feasible dual-template method. Ice and aqueous silica were used to tune macropores and mesopore sizes, while pyrolysis introduced micropores. To further enhance the internal electric field for efficient charge separation and to introduce a strain-induced electrical potential on the surface, we applied ultrasonic excitation to PbTiO_3_, which has both ferroelectric and piezoelectric properties [24]. More specifically, the localized quasi-1D nanoneedle morphology is beneficial for fast charge separation and increased delocalization of electrons [25, 26]. When compared with previous works, the architecture reported in this study has the following advantages: (1) The interconnected porous structure including macro-, meso-, and micropores provides sufficient space for light absorption, mass transfer, and ion trapping; (2) the pore size can be easily tuned and the two hard templates of ice and silica can be easily removed; (3) the pore structure has high thermal stability even at the annealing temperature of 900 °C, which is advantageous to retaining the appropriate pore combination, at the same time ensuring high carbon conductivity and high crystallinity of the metal oxides; and (4) this method can be extended to different ferroelectric and piezoelectric materials to design and fabricate unusual nano–nano or micro–nano composites for high-performance semiconductor photocatalysis. Our work thus improves understanding on the design of ferroelectrics-based photocatalysts and proposes a new strategy for the synthesis of metal oxide carbon-based composites with hierarchical trimodal pore distribution.

## Experimental Section

### Materials Synthesis

#### Chemicals

Titanium tetraisopropanolate (99%) and lead titanate (99%) were purchased from Aladdin Chemical Reagent Co., Ltd, China. Colloidal silica (30 wt.%) with particle dimension of 12 nm (Ludox, Sigma-Aldrich) was used as a hard template to introduce mesopores.

#### Preparation of PbTiO_3_/TiO_2_@C Composites

Titanium tetraisopropanolate was added dropwise into glacial acetic acid in the volume ratio 1:10, and the mixture was stirred for 24 h. The obtained white suspension was centrifuged and dried overnight at 65 °C; the obtained titanate precursor (TP) was water soluble. Next, TP (0.8 g), sucrose (0.32 g), and lead titanate were mixed under ultrasonic agitation with 1.1 g silica colloid (30 wt.%, 12 nm), 3 mL deionized (DI) water, TP, and sucrose. The amounts of lead titanate were adjusted according to the molar ratios between Pb and Ti of 1:1, 1:2, 1:4, 1:6, 1:8, and 1:10. The mixture was then immersed into liquid nitrogen for complete freezing and was freeze-dried for 10 h, then annealed at different temperatures in the range 600–1000 °C for 5 h in Ar atmosphere. The silica template was etched with 3 M NaOH at 80 °C for 48 h and then was washed with DI water for several times until neutral pH. Finally, the sample was dried under vacuum. Commercial P25 was used as the benchmark photocatalyst for comparison. PbTiO_3_/TiO_2_ (without addition of sucrose) and TiO_2_/C (without the addition of PbTiO_3_) were also used as controls.

### Characterization of Microstructure and Morphology

X-ray diffraction was performed with a diffractometer (XRD, PANalytical B.V., Netherlands) at the scanning range from 20° to 80°. The thermal stability was analyzed by TG–DTA (METTLER TOLEDO-3) at the heating rate of 10 °C min^−1^ under N_2_ flow. Fourier transform infrared spectroscopy (FT-IR, Perkin-Elmer 843) was used to study the surface molecular groups in the as-prepared samples. The molecular structural fingerprint was traced by Raman spectroscopy (LabRAM HR 800 microprobe spectrometer). Scanning electron microscopy [SEM, Ultra Plus, Carl Zeiss, Germany equipped with energy-dispersive spectroscopy (EDS)] was used to examine the surface morphology of the composites and determine the distribution of the different chemical elements in the composites. Transmission electron microscopy (TEM, JEM-2100) in both bright field and high-resolution modes was conducted to explore the detailed microstructure of samples. Selected area diffraction patterns were recorded to further investigate the microstructure. Information on surface area and pore structure was obtained by applying the Brunauer–Emmett–Teller (BET) method and Barrett–Joyner–Halenda (BJH) theory, respectively, and the pore size distribution was analyzed with the non-local density functional theory (NLDFT). By definition, micropores are pores with the sizes < 2 nm, while mesopores are in the size range of 2–50 nm and macropores have sizes > 50 nm. The electron binding energy of elements was analyzed through X-ray photoelectron spectroscopy (XPS, Thermo Fisher Scientific ESCALAB 250 and using monochromatic Al Kα radiation). UV–Vis diffuse reflectance spectroscopy (Lambda 35) was employed to characterize light absorption of the materials. Photoluminescence (PL) emission spectra were recorded with the help of HORIBA Fluoromax-4 spectrofluorometer with Xe lamp as the excitation source, and electron paramagnetic resonance (EPR) spectra were recorded at 77 K on a Bruker A300 spectrometer.

### Photocatalytic and Photoelectrochemical Setup

A 125-W high-pressure mercury lamp and a 300 UV Xe lamp (PLS-SXE 300) equipped with an ultraviolet cutoff filter (λ > 420 nm) were used as UV and visible light sources, respectively. For organic dye degradation, 80 mL of MB solution (5 × 10^−5^ mol L^−1^) was stirred for 30 min during which no auto-degradation of MB was observed. A known weight (15 mg) of the as-prepared photocatalyst powder was added into the MB solution and agitated for 30 min using a magnetic stirrer prior to irradiation, to enable the solution to reach adsorption/desorption equilibrium. Samples were withdrawn at regular intervals and analyzed after centrifugation to measure the optical density at the wavelength of maximum absorption of MB (664 nm). The irradiation intensities of UV and visible radiation were 310 and 250 W m^−2^, respectively. To investigate the influence of the piezoelectric field on the reactivity of PbTiO_3_-based photocatalysts, MB degradation was carried out while applying ultrasonic waves under visible light. During the whole experiment, the photoreactor was cooled by circulating water.

Photocatalytic water splitting was used to further evaluate the photochemical performances of the catalysts. To load the co-catalyst Pt, known amounts of H_2_PtCl_6_ along with 100 mg of the composite or P25 were added into 80 mL of a mixture of methanol and water (volume ratio = 1:3) and stirred for 1 h under UV irradiation. The photocatalysts were then washed with DI water and dried under vacuum at 70 °C for 10 h. Photocatalytic hydrogen generation was investigated with 100 mg of Pt-loaded samples dispersed in 100 mL of a water/methanol mixture (volume ratio = 4:1). The hydrogen content was measured by gas chromatography (Dongke, GC8890A) at regular time intervals. The apparent quantum efficiency (AQE) was measured under irradiation of a 300 W Xe lamp, and a 420-nm cutoff filter with a quantum meter was used to estimate the photon flux of the lamp. The following equation was used to calculate the AQE:

$${\text{AQE}} = 2 \times {\text{moles of hydrogen produced}}/h/{\text{moles of photon flux}}/h \times 100\%$$

**Scheme 1:**
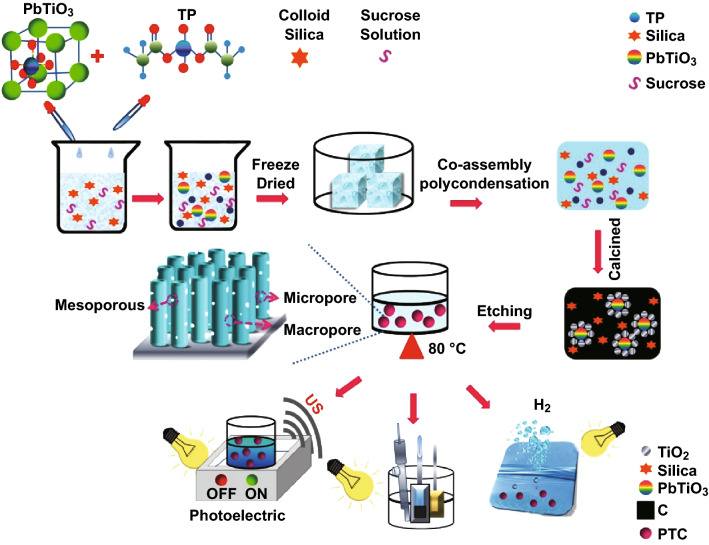
Schematic of the experimental setup showing the synthesis protocol and photochemical characterization

For photoelectrochemical performance measurements, the powder was mixed with N-methyl pyrrolidone and the mixture was coated on pre-cleaned FTO glasses (10 × 20 mm^2^) followed by vacuum drying at 80 °C, to form the working electrode. A saturated calomel electrode (SCE) served as the reference electrode, Pt was the counter electrode, and 1 mol L^−1^ Na_2_SO_4_ was the electrolyte. The photocurrent was measured at a scanning rate of 50 mV s^−1^ in the potential range 0–1.0 V under visible light irradiation. A 300-W high-pressure xenon lamp (420 nm cutoff filter) with the irradiance intensity of 250 W m^−2^ was selected as the irradiation source (Scheme 1).

## Results and Discussion

**Fig. 1:**
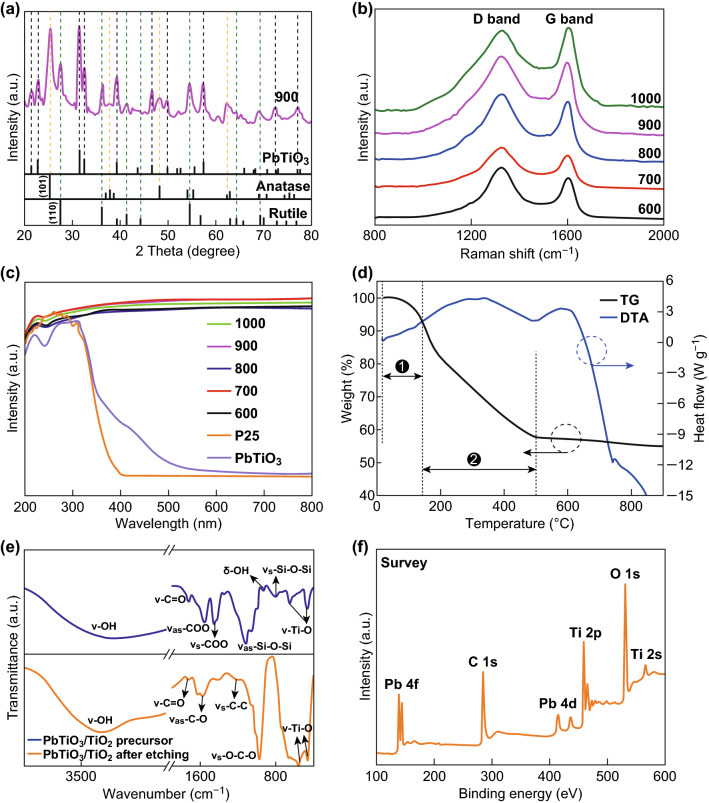
**a** XRD patterns, **b** Raman spectra, and **c** diffuse reflectance spectra of PTC annealed at different temperatures. **d** TG and DTA curves of the PTC precursor. **e** FT-IR spectra of PTC precursors after etching prior to annealing. **f** The complete XPS survey spectrum of PTC-900

The phase composition of the samples with a 1:8 molar ratio of PbTiO_3_ to TiO_2_ annealed at different temperatures from 600 to 1000 °C for 5 h (denoted as PTC-X; where X is the annealing temperature) was investigated by XRD; results are shown in Figs. 1a and S1. The peak locations of PbTiO_3_ (JCPDS No. 70-0746), anatase (JCPDS No. 21-1272), and rutile (JCPDS No. 21-1276) are also listed. All the observed peaks in the samples can be indexed to the above three structures, and no impurity phases are observed. The peak intensity increases with annealing temperatures, indicating that crystallinity of the as-prepared samples improved with temperature of annealing. For samples annealed at temperature > 600 °C, rutile peak is observed, indicating phase transformation from anatase to rutile [27, 28]. Anatase, which is generally considered have greater photoactivity in photochemical reactions, is effectively maintained in large amounts even at the annealing temperature of 1000 °C, although complete phase transformation from anatase to rutile usually occurs at 600 °C [28]. The high thermal resistance to phase transformation of the as-prepared PTC samples is thus beneficial to achieving high activity in photochemical reactions. The synergistic effect between anatase and rutile promotes electron transfer between the two phases and decreases the recombination rate of photogenerated charge carriers. This enhances the overall photochemical reactivity of biphasic TiO_2_ as compared with that with only single crystallographic phase [29, 30]. The high annealing temperature also led to coarsening of the crystals and increased crystallite size, (calculated using the Scherrer equation) as shown in Table 1; this is consistent with a previous report in literature [31]. Raman spectra of PTC composites annealed at different temperatures (Fig. 1b) are composed of two distinct peaks at 1324 and 1600 cm^−1^, which can be assigned to D and G bands of carbon, related to the defect/disordered *sp*^2^-hybridized C atoms and the *E*_2g_ graphitic mode of carbon, respectively [32, 33]. The integral intensity ratios (*I*_G_/*I*_D_), which generally indicate the extent of carbon graphitization, are calculated to be 1.212, 1.105, 1.055, 1.042, and 0.978 for samples PTC-1000, PTC-900, PTC-800, PTC-700, and PTC-600, respectively. In carbon-based metal oxide photocatalysts, a high conductivity is necessary to promote fast dissipation of electrons from the metal oxide so as to avoid the recombination of charge carriers. When coupled with metal oxides, carbon can also serve as a photosensitizer in transferring electrons between carbon and the metal oxide effectively widening the absorption bandwidth of the photocatalyst [34, 35]. This is consistent with UV–Vis diffuse reflectance spectral results (Fig. 1c) where all the samples showed absorption in the visible range due to the presence of carbon [36, 37]. PTC composites annealed at different temperatures show similar light absorption behavior; no visible light absorption is observed for P25, whereas the absorption edge of PbTiO_3_ is extended up to 550 nm [38]. This result is consistent with a previously reported observation that the presence of surface oxygen vacancies may introduce localized states in the band gap of PbTiO_3_, which can trap electron and holes and thereby extend absorption to lower energy regions [39–41]. The PL spectra of all the samples are shown in Fig. S2. The PL emission intensity of PTC-900 is the lowest among all the samples, indicating its low recombination rate for photogenerated electrons and holes [42]. The thermal stability of the PTC precursor was evaluated from TG/DTA curves. The total weight loss is distributed in three stages, as marked in Fig. 1d. The evaporation of adsorbed water and small organic molecules occurs in the temperature range between room temperature and 125 °C. Further heating to 500 °C primarily leading to around 40% weight loss of the sample, which is ascribed to crystallization and formation of anatase, is described by the broad exothermic peak observed in the temperature range 300–500 °C. The phase transformation from anatase to rutile occurs at around 600 °C [27, 28], as indicated by the DTA peak, and there is no weight loss above 500 °C; this result is consistent with XRD data. Organic groups present on the surface of the PTC precursor after etching prior to annealing were analyzed by FT-IR spectroscopy; results are shown in Fig. 1e. The deformation and vibration of –OH caused by intermolecular H bonds mainly appear at 3420 and 927 cm^−1^ [43], and the peak at 1706 cm^−1^ corresponds to the vibration of carbonyl and carboxyl groups (ν–C=O) [44]. Peaks at 1556 and 1445 cm^−1^ are related to asymmetric (*as*) and symmetric (*s*) stretching, indicating coordination bond formation in TiO_2_ [20]. One significant change in the FT-IR curves of unannealed PTC precursor after etching is the absence of peaks at 1122 and 766 cm^−1^, which are indexed to stretching vibration Si–O—originating from the SiO_2_ template. This result proves that SiO_2_ is completely eliminated by NaOH etching [45].

**Table 1:** Phase composition of anatase and rutile and the corresponding crystallite sizes in PTC composites annealed at different temperatures

Samples	Phase composition (%)	Crystallite size (nm)	
	Rutile	Anatase (101)	Rutile (110)
600	0	90.2	
700	0	102.4	
800	39.2	158.5	198.5
900	48.1	196.4	227.5
1000	54.5	208.6	276.4

Crystallite sizes of anatase and rutile were calculated using the (101) and (110) reflections

**Fig. 2:**
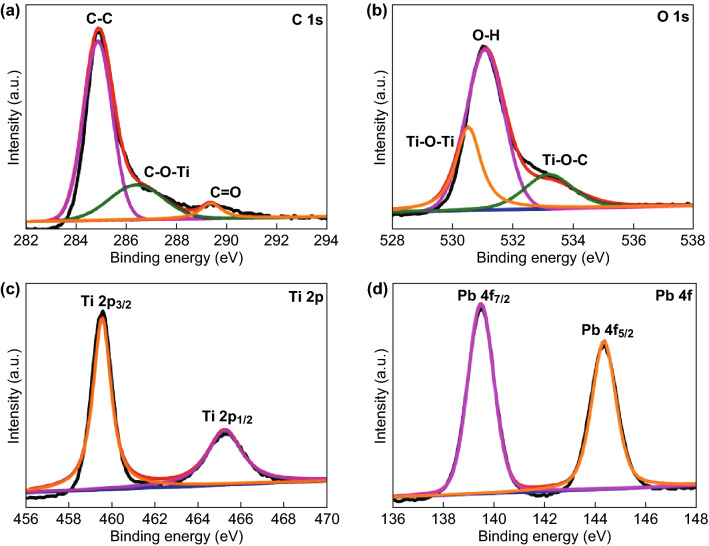
High-resolution XPS spectra of **a** C 1 s, **b** O 1 s, **c** Ti 2p, and **d** Pb 4f

Figure 1f presents the wide-scan XPS survey spectrum of PTC-900 in the binding energy range 100–700 eV. Only the four elements C, O, Ti, and Pb are detected. The deconvoluted spectra for each of these elements are presented in Fig. 2. In the C 1 s high-resolution spectrum (Fig. 2a), the three characteristic peaks at 284.8, 286.5, and 289.4 eV are assigned, respectively, to the binding energies of C–C, C–O–Ti, and C=O [46, 47], indicating that a chemical bridge for electron transfer is formed between carbon and TiO_2_. The O 1 s spectrum (Fig. 2b) can be divided into three peaks, Ti–O–Ti at 530.5 eV, O–H at 531.1 eV, and Ti–O–C at 533.2 eV [48, 49]. The Ti spectrum can be fitted to two peaks corresponding to Ti 2p_3/2_ and Ti 2p_1/2_, while the Pb spectrum consists of two peaks from Pb 4f_7/2_ and Pb 4f_6/2_ at 139.5 eV and 144.3 eV [50–53].

**Fig. 3:**
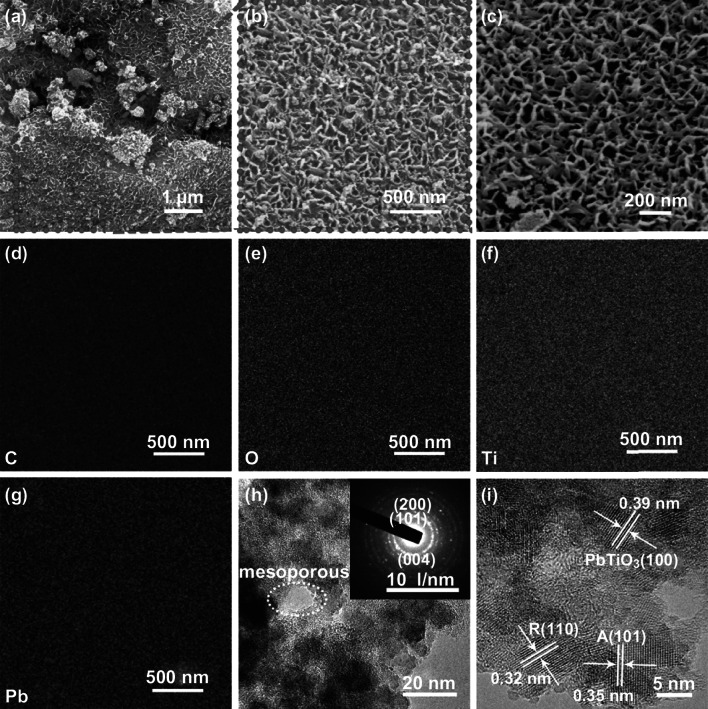
Detailed microstructure analysis of PTC-900 composites: **a–c** SEM images with different magnification, **d–g** EDX elemental mapping, **h** bright field TEM image with selected area electron diffraction (SAED) pattern shown in the inset, and **i** high-resolution TEM image with lattice fringes of PbTiO_3_ and TiO_2_ highlighted in the figure

The detailed microstructure and morphologies as well as pore structure of PTC-900 were analyzed from SEM to TEM images (Fig. 3). Field emission SEM images under different magnification (Fig. 3a–c) show an arrangement of porous walls with long range order surrounded by the nanoneedles of 10 nm diameter along with macropores with sizes in the 50–100 nm range. The local structure of the nanoneedle with high aspect ratio and a quasi-1D directional orientation is beneficial to the separation of charge carriers from the nanoparticles to the electrolyte due to the greater delocalization of electrons and the decreased charge carrier transfer resistance [25, 26]. The surrounding macropores provide channels for solution and electrolytes to access the inner surfaces of the composites materials and decrease the distance travelled by the charge carriers in photochemical reactions. In addition, the macropores around the nanoneedle can also cause light scattering within the interconnected framework and increase photon absorption of the composites. The corresponding SEM images are shown in Fig. S3 for reference. EDX (Fig. 3d–g) was conducted to check whether the constituent elements C/O/N/Pb are uniformly distributed in the composite; results show that this is the case and no hot spots are present. Bright field as well as high-resolution TEM images and the selected area electron diffraction (SAED) (Fig. 3h–i) provide further structural information on the composite. Micropores and mesopores can be clearly seen in the porous frame, and polymorphism of PbTiO_3_ and TiO_2_ is also confirmed. The crystalline lattice spacings are calculated to be 0.32, 0.35, and 0.39 nm, which are attributed to the (110) plane of anatase, (101) plane of rutile, and (100) plane of PbTiO_3_ [54].

**Fig. 4:**
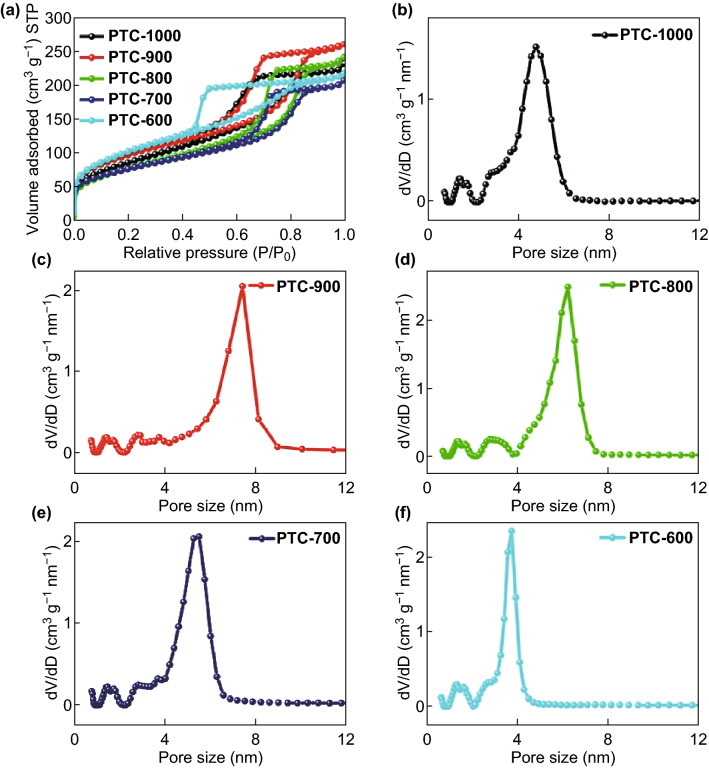
**a** Nitrogen adsorption–desorption isotherms and **b–f** the pore size distribution of PTC annealed at different temperatures in the range 600–1000 °C

The N_2_ adsorption/desorption isotherms and pore size distribution of PTC samples at different annealing temperatures are shown in Fig. 4 and Table 2. All the PTC composites show type IV isotherms due to capillary condensation in pores. Consistent with TEM results, the isotherms confirm the presence of mesopores. Pore size distributions of samples annealed at different temperatures show that primary pore size increased from 3 nm for PTC-600 to 5, 6, and 7 nm for PTC-700, PTC-800, and PTC-900; all the samples contain a certain percentage of micropores. The pore structure of PTC-1000 shows thermal condensation to 5 nm, indicating that the pore structure begins to collapse upon high temperature annealing. For carbon-based composites, high annealing temperatures are usually necessary to increase the graphitization degree as well as the crystallinity of metal oxide. One negative influence of high annealing temperature is the possible collapse of the pore structure. High thermal resistance is an advantage of our as-prepared PTC that maintains the pore structure intact even at the high temperature of 900 °C. According to Table 2, increasing the annealing temperature up to 900 °C leads to greater contributions from mesopores and micropores to the surface area, while the total surface area is lowered from 374.1 (600 °C), to 315.1 (900 °C) and then to 277.5 m^2^ g^−1^ (1000 °C). The mesoporous surface area of the composite annealed at 900 °C reaches the highest SSA value of 248.5 m^2^ g^−1^. Thus, trimodal pore size distribution of micropores, mesopores, and macropores is optimized.

**Table 2:** Surface area and pore structure of PTC composites annealed at different temperatures

Samples	S_BET_ (m^2^ g^−1^)	S_micro_ (m^2^ g^−1^)	S_meso_ (m^2^ g^−1^)	V_pore_ (m^3^ g^−1^)	V_micro_ (m^3^ g^−1^)	V_meso_ (m^3^ g^−1^)
600	374.1	30.1	152.9	0.296	0.013	0.256
700	353.3	32.4	184.8	0.309	0.026	0.269
800	325.5	43.7	234.5	0.358	0.031	0.319
900	315.1	54.2	248.5	0.389	0.038	0.342
1000	277.5	8.1	236.3	0.328	0.011	0.309

S_micro_ and S_meso_ are the surface areas of the micropores and mesopores, respectively; V_pore_ is the total pore volume; V_micro_ and V_meso_ are the volumes of the micropores and mesopores, respectively

**Fig. 5:**
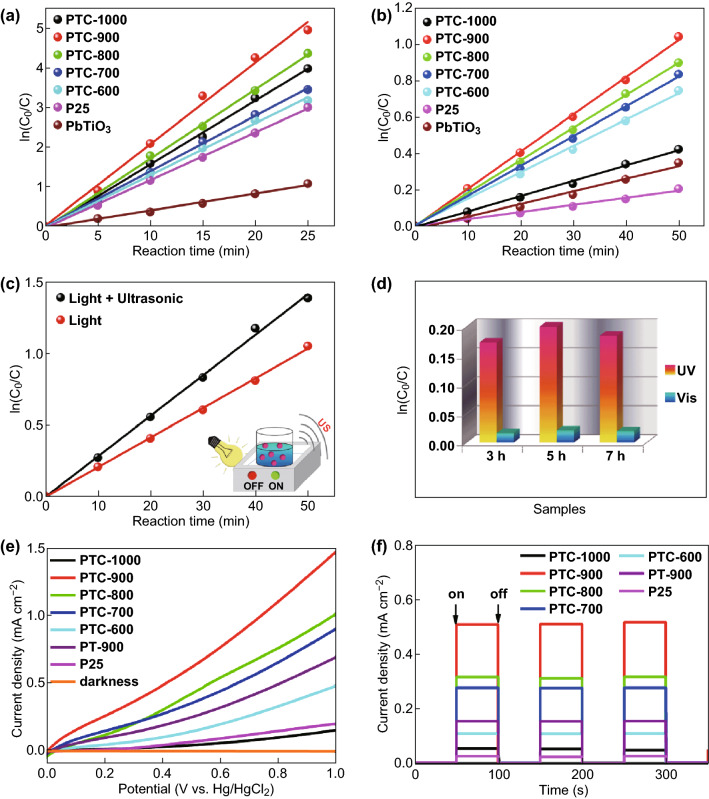
Photocatalytic MB degradation activity of PTC composites annealed at different temperatures under **a** UV, **b** visible, and **c** ultrasonic-assisted visible light irradiation. **d** Photocatalytic reactivity of PTC samples annealed at 900 °C for different annealing durations. **e** Photocurrent density against potential curves of PTC samples annealed at different temperatures. **f** Photocurrent response of PTC samples and P25 during repeated measurements

The photocatalytic activity of the composites for MB degradation under UV and visible light irradiation is shown in Fig. 5a, b. The degradation of MB is a first-order reaction, the rate of which is described by Eq. 1 [55]:

1 $${ \ln }\left( {C_{0} /C} \right) \, = {\text{ K}}t$$

where *C*_0_ is the initial concentration of MB, *C* is the concentration of MB at time *t*, *K* is the first-order reaction rate constant, and *t* is the reaction time. All reaction curves show the excellent linear fitting to the first-order kinetics with *R*^2^ values > 0.998. Increasing the annealing temperature from 600 to 900 °C leads to enhanced reactivity, but further annealing to 1000 °C causes a dramatic decrease in reactivity. Results for TiO_2_, PbTiO_3_/TiO_2_ (PT-900), and TiO_2_/C (TC-900) annealed at 900 °C are also listed for reference (Fig. S4), where it is seen that reactivities under both UV and visible light irradiation are much lower than that of PTC-900. The annealing temperature can influence the pore structure (Table 2), but an appropriate combination of pores of different dimensions is required for optimal performance. High micropore and mesopore surface area can provide abundant active sites for redox reactions and promote the diffusion of reagents to the inner surface of catalysts to enhance the accessibility of active sites [56]. Moreover, high annealing temperatures increase the graphitization degree of the carbon coating (Fig. 1) which helps in the timely transfer of electrons from the semiconductor to carbon. The quality of the interfaces between the different phases can also be improved during annealing by forming strong chemical bonds to decrease charge carrier resistance. However, very high annealing temperature can lead to a dramatic decrease in surface area, collapse of the pore structure and also to phase change of TiO_2_, all of which can adversely affect photocatalytic reactivity. Hence, the reactivity of PTC-1000 is reduced.

All the PTC samples show higher photocatalytic decomposition rate of organic dyes than both commercial TiO_2_ (P25) and pure PbTiO_3_ under both UV and visible light irradiation. The corresponding optical images of MB degradation with PTC-900 are illustrated in Fig. S5. The MB degradation rate of PTC-900 is approximately 7.2 (UV)/3 (Vis) times the corresponding values for PbTiO_3_. Considering that carbon is photocatalytically inert but contributes to the total mass, the actual reactivity enhancement for PTC samples over PbTiO_3_ and TiO_2_ is even higher. Other processing parameters including Pb/Ti ratio as well as annealing time have also been optimized as shown in Fig. 5d, S6, and S7, and the list of K values in Tables S1–S3. The EPR spectrum of PTC-900 is shown in Fig. S8, where both lattice electron trapping sites for anatase (*g* value of 1.999 and 1.954) and rutile (*g* value of 1.975 and 1.939) are observed. A distinct signal at the *g* value of 2.003–2.006 is also detected, which we attribute to an oxygen-centered radical related to surface-trapped holes [57]. Our previous work on PbTiO_3_, one of the most important piezoelectric semiconductors, has shown that the internal electric field within the ferroelectric can create a charged surface to mitigate the recombination of photogenerated electrons and holes [3, 8, 10, 12, 58]. Herein, we have verified if an externally applied mechanical field on piezoelectric PbTiO_3_ can further enhance the photochemical activity of the composite. As shown in Fig. 5c, we applied ultrasonic waves during irradiation to induce an external mechanical field and found that the reactivity of PTC under irradiation (UV/Vis) is approximately 1.4 times that of the untreated sample. Samples annealed at other temperatures (Table S2) also showed similar enhancement of 1.5–2.3 times with respect to the corresponding control samples. This enhancement may originate from the piezoelectric electric field that can assist in improving the photocatalytic activity of PTC samples. In addition, the cavitation during sonication could eliminate the energy barrier for gas bubble nucleation, which is also favorable to increasing reactivity. In addition to dye degradation, the photochemical reactivity of the prepared PTC samples was further investigated under visible light irradiation in the presence of an applied potential in the range 0–1.0 V versus SCE [59, 60]. Consistent with results of photocatalytic dye degradation, PTC-900 shows the highest photocurrent density of 1.48 mA cm^−2^ among all the samples tested (Fig. 5e), while the readout value for PTC-600, PTC-700, PTC-800, and PTC-1000 is 0.48, 0.91, 1.01, and 0.15 mA cm^−2^, respectively. The dark current density remained at a very low value (< 0.001 mA cm^−2^) in all the cases. PTC samples at the bias voltage of 0.4 also show the excellent photostability (Fig. 5f) without no apparent photocurrent density loss under visible light irradiation.

**Fig. 6:**
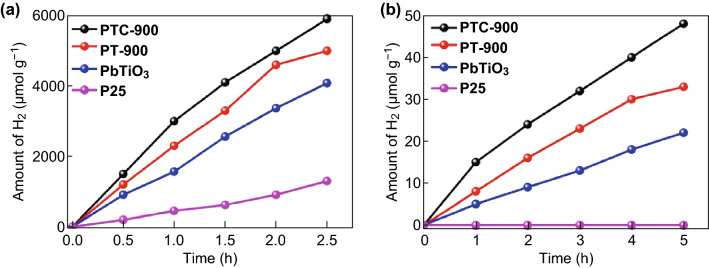
Photocatalytic hydrogen production of different photocatalysts loaded with 2% Pt as co-catalyst under **a** UV and **b** visible light irradiation

PTC-900 and its components were also tested in the water-splitting process for hydrogen generation under both UV and visible light irradiation (Fig. 6a, b). When loaded with 2 wt.% Pt, the hydrogen production rate for PTC-900 was approximately 2360 μmol h^−1^ g^−1^ under UV radiation, while the corresponding values for pure PbTiO_3_ and P25 were 520 and 1632 μmol h^−1^ g^−1^, respectively. PTC-900 also shows outstanding hydrogen generation under visible light irradiation with a generation rate of 9.6 μmol h^−1^ g^−1^, which is much better than that observed for PbTiO_3_. No hydrogen production was detected for P25 under visible light; the hydrogen production for PT-900 is also listed for reference. The AQE of the as-prepared PTC sample is calculated to be − 0.32% at 420 nm. In addition, the influence of different co-catalyst loading amounts on the photocatalytic activity is showed in Fig. S9, where it is seen that 2 wt.% Pt-PTC shows the highest hydrogen production rate. The photocatalytic stability for dye degradation and hydrogen production of PTC-900 have also been evaluated with results shown in Fig. S10. After four cycles, no apparent decay in photocatalytic reactivity and photocatalytic is observed, indicating the excellent photostability of the as-prepared samples [61–64].

**Fig. 7:**
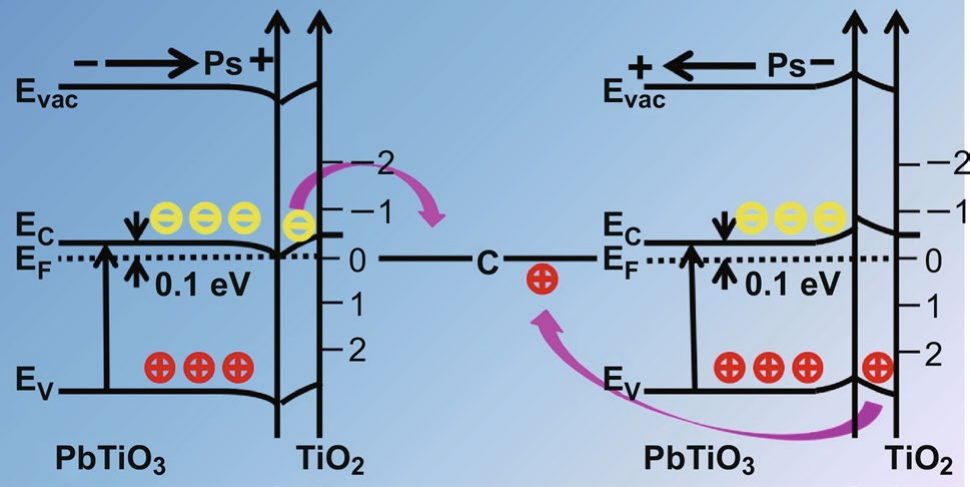
Schematic of the energy level diagram for the band structure of PbTiO_3_-TiO_2_ system assuming different polarization (Ps) directions. *E*_c_, *E*_v_, *E*_f_, and *E*_vac_ are the energies of the conduction band, valance band, Fermi level, and vacuum level

Herein, we attribute the enhancement of photochemical reactivities of semiconductor photocatalysis to the combination of two effects: materials design and microstructure optimization. Based on our previous work on the role of internal electric field on photocatalytic activity [3–5], we designed a carbon-based semiconductor heterostructure junction to combine piezoelectric photocatalysis with carbon to improve the charge transfer between the different phases by increasing electrical conductivity. A highly simplified energy level diagram (shown in Fig. 7) roughly illustrates possible band bending between TiO_2_ and PbTiO_3_ to highlight the influence of the electric field on charge carrier transfer between the photoactive components; details with respect to interaction with carbon are not shown. The pH value is also as assumed to remain constant throughout the reaction. The polarization of the specified interface is taken as “positive” and the opposite direction as “negative.” The band in the space charge region is bent to separate charge carriers. The internal dipolar field induced from ferroelectricity can direct the transfer of the photogenerated electrons and holes in different directions thus separating the charge carriers [65–67]. The positive polarization can reduce band bending in PbTiO_3_ compared with the neutral situation (without any electric field) and electrons preferentially transfer to TiO_2_. Correspondingly, a negative polarization will increase band bending to promote holes transfer [3]. In the current case, a high annealing temperature is required to build a strong interface so as to decrease charge carrier transfer resistance between the different phases. For piezoelectric PbTiO_3,_ in addition to the ferroelectric dipolar field, the electric field is also enhanced under the externally applied mechanical wave. Correspondingly, mechanically assisted photocatalysis is effective, as demonstrated by all the PTC samples showing enhanced reactivity under ultrasonic treatment.

To tailor the microstructure, a novel dual templating method was developed resulting in PbTiO_3_/TiO_2_@C composites with hierarchical pore structure including pore sizes at three length scales (micro-, meso-, and macropores). Ice and silica were used as hard templates to introduce macropores and mesopores. The pore size can also be tuned by changing the annealing temperature. Moreover, the structure with quasi-1D nanoneedles on specific locations is also beneficial for separating the charge carriers by increasing the number of charge carriers at the surface to participate in redox reactions.

## Conclusions

In the current work, PbTiO_3_/TiO_2_@C composites with hierarchical porous structure with pore sizes on the micro-, meso-, and macro- scale were synthesized via a novel dual templating method. Localized nanoneedle structures surrounded by macropores were observed. The as-prepared composites showed excellent photocatalytic performance for both organic pollutant degradation and hydrogen production by water splitting. The hydrogen generation rate of the best PTC sample was as high as 2360 and 9.6 μmol h^−1^ g^−1^ under UV and visible light irradiation; these values are much better than those of pure P25 and PbTiO_3_. A high photocurrent density of 1.48 mA cm^−2^ at the potential of 1.0 V versus SCE was also obtained. More importantly, the photocatalytic activities of PTC samples were further enhanced by a factor of 1.5–2.3 under ultrasonic treatment. The outstanding performance of PTC samples can be attributed to their unique pore structures, large surface area as well as the high graphitization degree of the carbon coating. The present work is important in developing piezoelectricity-assisted photocatalytic behavior and provides new routes to design high-performance photocatalyst materials targeted toward energy and environmental applications.

## Electronic supplementary material

Below is the link to the electronic supplementary material.

Supplementary material 1 (PDF 770 kb)
