# Supplementary material for: Ferroelectric Oxide Nanocomposites with Trimodal Pore Structure for High Photocatalytic Performance
Source: Nanomicro Lett. 2019 May 4;11:37. doi: 10.1007/s40820-019-0268-y (PMC7770796; doi:10.1007/s40820-019-0268-y)
Supplement: Supplementary file 1 — Supplementary material 1 (PDF 770 kb) [file 40820_2019_268_MOESM1_ESM.pdf]

Supporting Information for

# Ferroelectric Oxide Nanocomposites with Trimodal Pore Structure for High Photocatalytic Performance

Tingting Xu[1], Xuan Liu[1,] *, Shulan Wang[1,] *, Li Li[2,] *

[1]Department of Chemistry, School of Science, Northeastern University, Shenyang 110819, People's Republic of China

[2]School of Metallurgy, Northeastern University, Shenyang 110819, People's Republic of China

*Corresponding authors. E-mail: xuanliucmu@gmail.com (Xuan Liu); slwang@mail.neu.edu.cn (Shulan Wang); lilicmu@alumni.cmu.edu (Li Li)

## Supplementary Figures and Tables

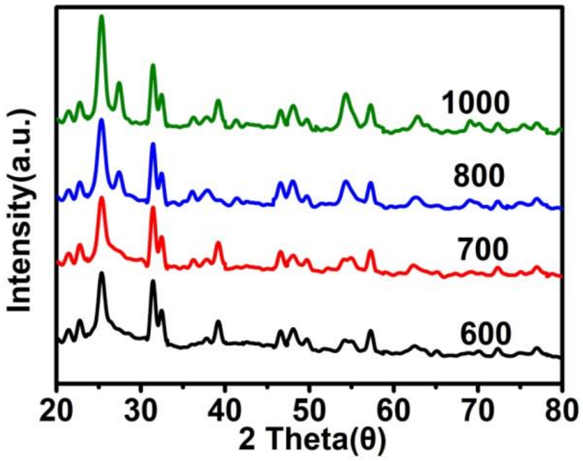


**Fig. S1** XRD diffraction patterns of PTC samples annealed at different temperatures (PTC-600, 700, 800, and 1000)

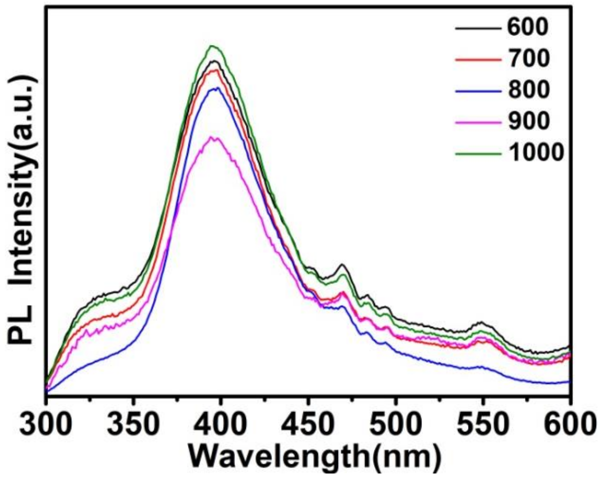


**Fig. S2** PL emission spectra of PTC samples annealed at different temperatures

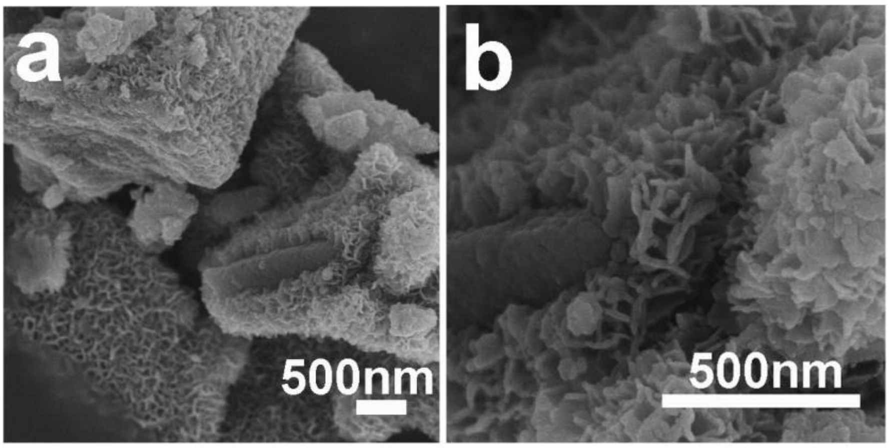


**Fig. S3** SEM images of **a** $PbTiO_3/TiO_2$ and **b** $TiO_2$

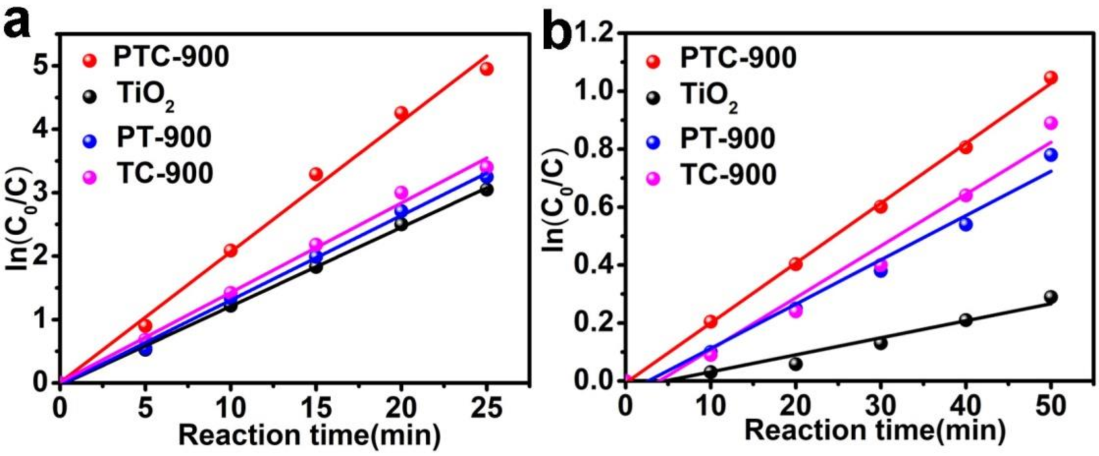


**Fig. S4** Photocatalytic MB degradation of PTC-900, $TiO_2$, PT-900, TC-900 annealed at 900 °C under **a** UV and **b** visible light irradiation

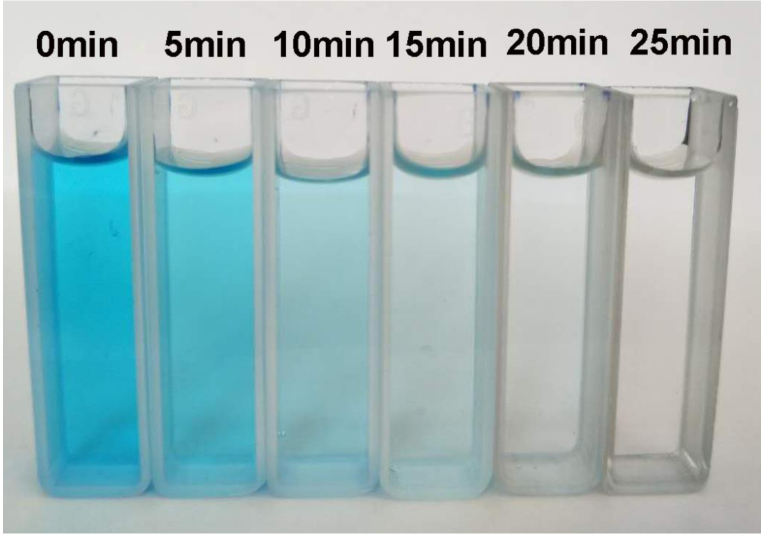


**Fig. S5** The optical image of MB degradation with PTC-900 under UV exposure to illustrate the photocatalytic organic dye degradation

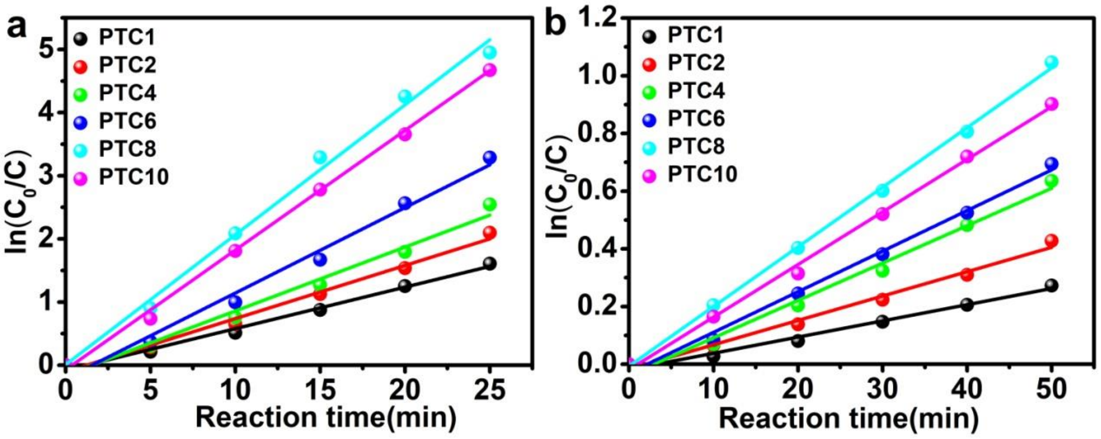


**Fig. S6** Photocatalytic MB degradation of PTC samples with different ratios of $PbTiO_3$ to $TiO_2$ under **a** UV and **b** visible light irradiations

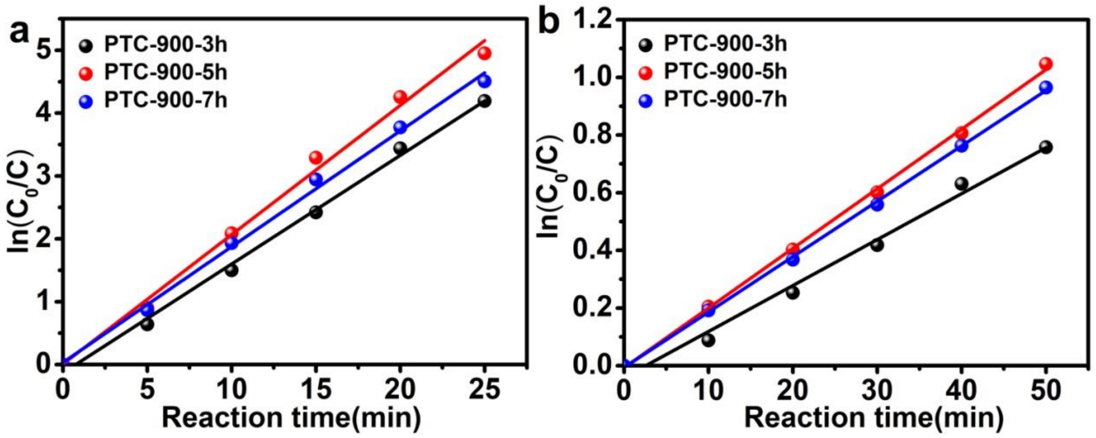


**Fig. S7** Photocatalytic MB degradation of PTC nanocomposites annealed at 900 °C for different time under **a** UV and **b** visible light irradiation

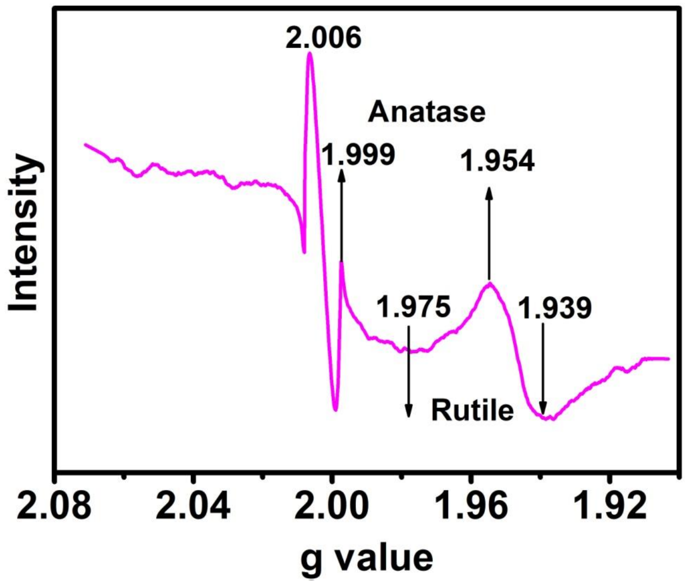


**Fig. S8** EPR spectrum of PTC-900 with the detailed g value marked

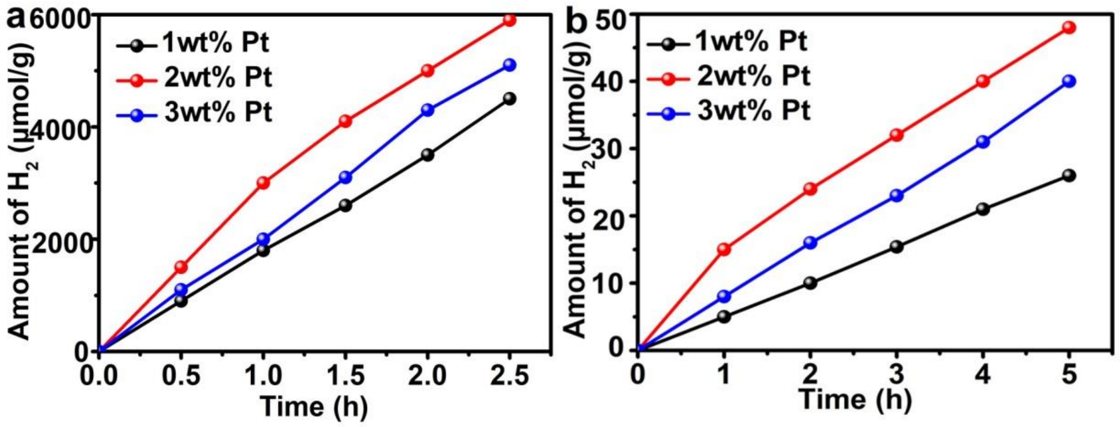


**Fig. S9** Photocatalytic hydrogen production of PTC-900 loaded with 1%-3% Pt as co-catalyst under **a** UV and **b** visible light irradiation

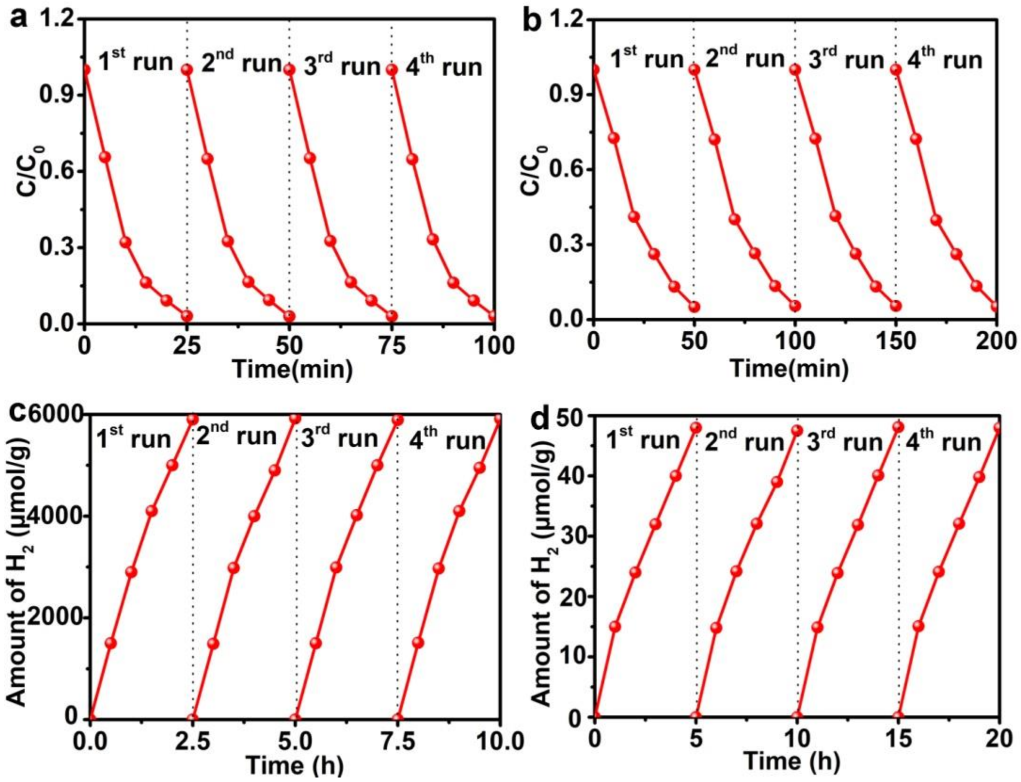


**Fig. S10** Photocatalytic stability of PTC-900 for **a, b** MB degradation and **c, d** hydrogen production (loaded with Pt as co-catalyst)

**Table S1** *K* values of PTCx nanocomposites annealed at 900 °C for 5 h with different molar ratios of $PbTiO_3$ to $TiO_2$ for MB degradation under UV and visible light irradiation

| Slope ($min^{-1}$) | $K_{UV}$ | $K_{vis}$ |
|---|---|---|
| PTC1 | 0.06576 | 0.00562 |
| PTC2 | 0.08417 | 0.00845 |
| PTC4 | 0.10121 | 0.01296 |
| PTC6 | 0.13532 | 0.01408 |
| PTC8 | 0.20577 | 0.02068 |
| PTC10 | 0.18907 | 0.01823 |

**Table S2** *K* values of PTC nanocomposites with 1:8 mole ratio of $PbTiO_3$ to $TiO_2$ annealed for 5 h at different temperatures for MB degradation under UV, visible light irradiation and ultrasonic assisted visible light irradiation

| Slope ($min^{-1}$) | $K_{UV}$ | $K_{vis}$ | $K_{vis}$ (US) |
|---|---|---|---|
| 1000 | 0.16096 | 0.00851 | 0.01981 |
| 900 | 0.20577 | 0.02068 | 0.02827 |
| 800 | 0.17481 | 0.01809 | 0.02559 |
| 700 | 0.13978 | 0.01655 | 0.02424 |
| 600 | 0.13107 | 0.01463 | 0.02208 |
| P25 | 0.12040 | 0.00393 | - |
| $PbTiO_3$ | 0.04249 | 0.00701 | - |

**Table S3** *K* values of PTC nanocomposites annealed at 900 °C for different annealing time for MB degradation under UV and visible light irradiation

| Slope($min^{-1}$) | $K_{UV}$ | $K_{vis}$ |
|---|---|---|
| PTC-3h | 0.17298 | 0.01594 |
| PTC-5h | 0.20577 | 0.02068 |
| PTC-7h | 0.18428 | 0.01922 |
